# Cellular reprogramming as a tool to model human aging in a dish

**DOI:** 10.1038/s41467-024-46004-5

**Published:** 2024-02-28

**Authors:** Patricia R. Pitrez, Luis M. Monteiro, Oliver Borgogno, Xavier Nissan, Jerome Mertens, Lino Ferreira

**Affiliations:** 1 Center for Neuroscience and Cell Biology, University of Coimbra, Coimbra, Portugal; 2 Faculty of Medicine, University of Coimbra, 3000-548 Coimbra, Portugal; 3 IIIUC-institute of Interdisciplinary Research, University of Coimbra, Casa Costa Alemão, Coimbra, 3030-789 Portugal; 4 Department of Neurosciences, University of California San Diego, La Jolla, CA USA; 5 Laboratory of Genetics, The Salk Institute for Biological Studies, La Jolla, CA USA; 6 CECS, I-STEM, AFM, Institute for Stem Cell Therapy and Exploration of Monogenic diseases, Evry cedex, France

**Keywords:** Stem-cell biotechnology, High-throughput screening, Tissue engineering, Reprogramming, Biomedical engineering

## Abstract

The design of human model systems is highly relevant to unveil the underlying mechanisms of aging and to provide insights on potential interventions to extend human health and life span. In this perspective, we explore the potential of 2D or 3D culture models comprising human induced pluripotent stem cells and transdifferentiated cells obtained from aged or age-related disorder-affected donors to enhance our understanding of human aging and to catalyze the discovery of anti-aging interventions.

## Abstract (web summary)

The development of human cellular models of aging that surpass the limitations of animal models of aging is urgent. Here, the authors explore the opportunities and limitations of cellular reprogramming to create reliable aging in vitro models and their potential for the discovery of anti-aging compounds.

## Introduction

The generation of human model systems is critical to reveal the mechanisms underlying the aging processes and to gain insights on interventions that extend the human organism’s health/life span. The use of non-vertebrate (yeast, worm, and fly) and vertebrate (zebrafish, mice, primates) models have already provided valuable insights on key proteins/pathways governing organismal aging (e.g., insulin/IGF-1, mTOR, AMPK, and sirtuins) and how their manipulation impacts health/life span^1–3^. Although these models can reproduce aspects of human aging and aging-associated diseases, they also have several limitations. First, non-vertebrate models have limited capacity to recapitulate the human complexity, in that they lack organs and systems that are important in human aging, such as a closed circulation system and an adaptive immune system. Second, the life span and rate of aging among most vertebrate species used in aging studies dramatically differs from humans^4^ and inbred and genetic models typically fail to account for the biological variability that exists amongst humans^5^. Although the basis for these differences remains largely unknown, it is likely that human-specific changes in epigenetics (e.g. DNA methylation), telomere length, protein composition, among others, contribute to those differences, and thus demand human models to complement our understanding. Third, although age-related diseases such as progeroid syndromes^6^, Parkinson´s disease (PD), Alzheimer´s disease (AD) may be an inspiration to elucidate the biology of aging, transgenic animal models do not fully recapitulate human age-related diseases with high fidelity, and can be expected to only partially predict the interface between aging and disease pathways.

Human cell models, such as primary cell samples isolated from young and old individuals, or from individuals affected by age-related diseases, can be a platform to study aging in a dish^1^. Yet, their limited availability (requires the participation of clinically/physically well-characterized human donors), inaccessibility of some tissues (e.g. brain, heart, blood vessels) and their finite in vitro expansion capacity (Hayflick limit) are major hurdles. Moreover, some primary cellular models fail to recapitulate some important aging aspects at tissue or organ levels because (i) the cells change phenotype once cultured in vitro, (ii) model cell lines are established by a small number of founding cells, and (iii) some crucial 3D spatial cell-cell interactions are absent^7^.

In this Perspective, we discuss the opportunities and limitations of using human induced pluripotent stem cells (iPSCs) and transdifferentiated cells to complement animal models, and in what aspects they might be better suited to understand the human aging process. Specifically, we discuss how reprogramming-based cell models from aging donors and patients with genetic and sporadic age-related diseases, including both iPSC-based and directly transdifferentiated cells, can advance our understanding of the human aging processes and help with the identification of anti-aging drugs. We emphasize recent progress in this area^8–16^ in which human cell models have proven to be very valuable for aging research as well as drug screening. These patient-specific cellular models often better reflect the dynamics and heterogeneity of aging processes across different human individuals whilst recapitulating the complex organization of tissue/organs at an unprecedented level. Finally, because cellular models are amenable to gene editing technologies, we explore how the introduction or correction of variants associated with aging-related disorders benefits current cell modeling research on aging.

## From aging molecules to individuals, and aging-associated diseases

Aging is a naturally occurring complex process involved in every biological and physiological system. On the molecular level, progressive aging is associated with the accumulation of molecular damage and functional alterations. Examples include the accumulation of mutations in the DNA sequence and detrimental post-translational protein modifications (e.g. oxidation) that lead to protein misfolding, dysfunction of molecular pathways, and a gain of aberrant or toxic protein activity^17^. On a cellular level, persisting molecular damages can cause a decline in cellular functionality, impair the regenerative capacities of cells, and make individual cells more prone to death or to regressing towards cellular senescence^18,19^. In this Perspective, aged cells are referred to cells that present at least one of the nine hallmarks that represent common denominators of aging in different organisms such as genomic instability, telomere attrition, epigenetic alterations, loss of proteostasis, deregulated nutrient sensing, mitochondrial dysfunction, cellular senescence, impaired stem cell fate and altered intercellular communication^19^. Loss of functional cells or the accumulation of malfunctioning and senescent cells can disrupt tissue and organ homeostasis and function. For example, senescent cells signal to their environment and alter the physiologic properties of surrounding cells, affecting their function, and altering the physiology of entire organs. Thus, aged tissues and organs usually not only perform less optimally but are also more vulnerable to develop chronic aging-related diseases (loss of resilience). On the level of an aging human individual, aging is typically perceived via phenotypic changes like for example graying of hair, wrinkling of skin or forgetfulness. However, for each individual person aging may manifest in different ways because different organs age at different biological rates^20^, dictating a higher incidence of developing aging-related diseases. Importantly, aging-associated diseases must be distinguished from impairments associated with normal aging, because lower cardiovascular fitness and a decline in cognitive abilities are normal, while myocardial infarction, AD, or cancer cannot be considered normal^21^.

In fact, aging is associated with a broad spectrum of different diseases, and one approach to establish a categorization of aging-associated diseases is by classifying them based on their interaction with normal biological aging, e.g. if (i) aging is primarily a major risk factor for the disease, (ii) if the disease causes pre-mature aging symptoms, or (iii) if the disease accelerates physiological categories of aging.

*Diseases with aging as a major risk factor* are characterized by increasing disease incidence with advancing age. The prevalence of cardiovascular diseases, including hypertension, coronary heart disease, heart failure, and stroke, increases from about 40% in men and women aged 40–59 years to 70–75% in those aged 60–79 years as they constitute the major cause of death of the elderly population^22^. Further, more than a third of new cancer cases occur in people aged 75 and over, with the highest age-specific incidence rates being at 85 to 89 years of age^23^. Cells that undergo cancer-like transformations bypass the inherent cellular programs that prevent such changes, and aging is the most important risk factor for cancer. Genetic mutations play a crucial role in cancer development, but the mere accumulation of DNA mutations over time is insufficient to explain this relationship, but rather failing safeguarding mechanisms such as impaired DNA damage repair mechanisms and failing immune pathways to prevent tumor development contribute to this relationship^24^. Nearly 60 years ago, Hayflick and Moorhead showed that cells cultured in vitro have a finite capacity for cell division^25^. They also noted that cultured cells enter a state of irreversible cell growth arrest, a process that they called replicative senescence. Cellular senescence is a major pathway suppressing the initiation of cancerous transformation, but already Hayflick suggested that replicative senescence contributed to organismal aging. Further, senescence can accelerate cancer progression once initiated through the senescence-associated secretory phenotype (SASP) that senescent cells utilize to mobilize the immune system^26^. Accumulation of cellular senescence is therefore proposed as a major link between aging, the immune system, and the progression of aging-driven diseases^18,19^. Several independent lines of research identified aging-related changes in the immune system to be associated with different diseases, constituting a major risk factor across the human body, and coined terms such as ‘inflammaging’ and ‘immunosenescence’^24,27^.

*Diseases that cause premature aging phenotypes* are markedly distinct from age-dependent diseases, and encompass several progeroid syndromes. The word “progeroid” comes from the Greek words “pro” (before, premature) and “gēras” (old age). Hutchinson-Gilford progeria syndrome (HGPS) is the most prominent and most prevalent form of progeroid syndromes. Patients with HGPS have accelerated markers of body-wide aging signatures (skin, cardiovascular, etc.) and they often die by heart attack or stroke^28,29^. HPGS is caused by a mutation in the LMNA gene, which interferes with canonical splicing of the gene and leads to a truncated form of the protein called progerin, while under normal conditions lamin A, the protein product of LMNA, is farnesylated and cleaved by a metallopeptidase^30^. Non-farnesylated progerin accumulates and thickens the nuclear lamina, leading to a loss of peripheral heterochromatin and dysfunctional nuclear pores, and loss of non-homologous and homologous DNA-repair cascades. The premature aging phenotypes may result from the destabilization of the nuclear lamina and impaired DNA-repair mechanisms, which are also critical in other premature aging diseases such as Werner syndrome^31^.

*Diseases that accelerate measures of normal physiological aging* have gained the attention of the aging research field more recently. This development was sparked by the novel ability to quantitatively measure the biological aging process through imaging, fluid biomarkers, and the discovery of epigenetic clocks^32,33^. With the advent of quantitative biological aging technologies, neuropsychiatric diseases such as post-traumatic stress disorder, major depressive disorder, schizophrenia, and bipolar disorder have been shown to accelerate the rate of physiological aging in affected individuals^34,35^. These psychiatric disorders cause a low level of chronic inflammation, the accumulation of oxidative stress markers, accelerated epigenetic aging and a decrease in the amount of mitochondrial genome copy numbers^36^. A more comprehensive understanding of the molecular-level relationships between neuropsychiatric conditions and ’classical’ aging-related neurodegenerative disorders is imperative, not only to enhance our comprehension of neuropsychiatric risk factors for aging-related neurodegenerative disorders, but also to benefit patients with neuropsychiatric conditions as they age more rapidly.

## Modeling human aging with cell reprogramming models

### Human iPSCs

Human iPSCs can provide a continuous supply of tissue-specific cell types, which can be issued to monitor aging processes using different strategies^37^. Interestingly, advanced human age does not impair the reprogramming of somatic cells into iPSCs^38^, and iPSCs generated from centenarians showed gene expression profiles and differentiation potentials similar to human embryonic stem cells^39^. While iPSCs are now largely considered to be globally rejuvenated, it is interesting to note that early-passage iPSCs retain the minimal but detectable epigenetic signature of aging that however diminishes quickly with passaging^38^. In the big picture however, the rejuvenation process that takes place during the reprogramming of old cells already during the early stages of iPSC reprogramming constitutes a limitation for using iPSC-based models to capture aging signatures of primary cells, e.g. from old donors^40,41^. However, many 2D and 3D protocols for a variety of cell type- and tissue-specific differentiation protocols are available for iPSCs, and they are very amenable to genetic manipulation. Thus, and despite their lack of endogenous donor-specific aging signatures, this outstanding versatility allows for mechanistic studies in iPSC relating to aging, such as how certain genes and pathways influence cellular aging starting from zero. During recent years several approaches have been explored to induce an aging phenotype in rejuvenated iPSC models, and we summarize some of these in the section below.

#### Inducing aging in non-diseased iPSCs-derived cells

**Fig. 1:**
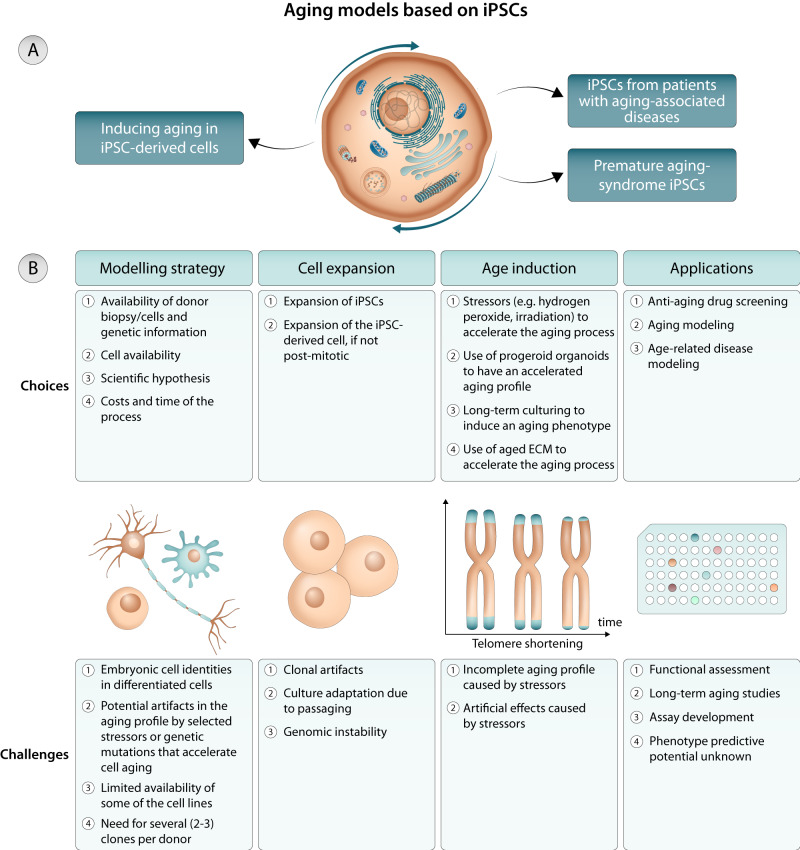
Modeling human aging with iPSCs. **A** Strategies to model human aging by (i) inducing aging in iPSC-derived cells, (ii) iPSCs from patients with aging-associated diseases and (iii) premature aging-syndrome iPSCs. **B** Choices and challenges in the use of iPSCs for human aging modeling.

Several strategies have been developed to induce an aging phenotype in wild-type iPSC-derived cells including: (i) long-term culture^42–44^, (ii) culture in aged extracellular matrix (ECM)^11^, (iii) telomere shortening^41^, and (iv) CRISPR/Cas9 technology^45^ (Fig. 1). These strategies have been used to age cardiomyocytes (CMs)^11,42^, neurons^43^ and mesenchymal stromal cells (MSCs)^10^. In vitro aging by long-term culture of cells was performed between 3^10^ and 12 months^43^, and in some studies was done by culturing the iPSC-derived cells in aged ECM (e.g. decellularized ECM from 22–24 months old mice) for 3-4 months^11^. These strategies resulted in the appearance of age-associated markers, including a decrease in cell proliferation^10^, an increase in DNA damage and mitochondrial reactive oxygen species (ROS) generation^41^, high expression of senescence-associated genes^10^ and proteins (p21^42^ and SA-β-galactosidase^10,42^), DNA methylation changes at senescence-associated CG dinucleotides^10^, increase in lipofuscin accumulation^11^, among others. This indicates that long-term culture can successfully produce an at least partially aged phenotype in iPSCs-derived cells, and such iPSC-derived accelerated aging models were instrumental to: (i) investigate the metabolic changes occurring during replicative senescence^10^, (ii) study the role of ECM in inducing an aged phenotype^11^, (iii) dissect the role of senescence in post-mitotic cells^42^ and (iv) study the phenotype of aged midbrain dopamine neurons^41^. Because these aging models rely on the induction of aging by stressors that might not occur in physiologic conditions, their direct biological relevance to human aging, and as a risk factor for age-related disorders, remains limited (Fig. 1).

#### iPSCs from aging-associated diseases

Human iPSCs carrying genetic mutations that occur in age-related diseases may provide invaluable information regarding the mechanisms governing aging. This has been explored in iPSC-derived CMs (reviewed in ref. ^46^) and iPSC-derived neurons (reviewed in ref. ^47^). For example, it has been shown that iPSC-derived CMs from individuals with end-stage hypertrophic cardiomyopathy or dilated cardiomyopathy aged faster than wild type iPSC-derived CMs as confirmed by significant shorter telomeres^48^. The accelerated aging profile in iPSC-derived CMs from individuals with dilated cardiomyopathies may be due to a dysfunctional activation of the platelet-derived growth factor signaling pathway^49^. Notably, most studies using iPSC-based neuronal models that carry disease-causing mutations applied additional stressors in tissue culture to elicit phenotypes, likely because rejuvenated iPSC-based models are young and fit, and thus do not develop pathologies spontaneously within reasonable culture periods of less than a few months to a year. Examples of such aging-related stressors include H_2_O_2_, 6-hydroxydopamine (6-OHDA), valinomycin, or carbonyl cyanide *m*-chlorophenyl hydrazone (CCCP) in iPSC models for PD^50–52^, proteasome inhibition in Huntington’s disease neurons^53^ or excitotoxicity in Ataxia^54^ (Fig. 1). Cocktail of stressors have been also used to induce senescence in neurons derived from iPSCs of amyotrophic lateral sclerosis (ALS) patient^55^. However, studies also report spontaneous disease phenotypes in iPSC models under standard culture conditions, which are likely downstream features of the mutated genes such as the constitutive active PD-causing LRRK2 kinase^56,57^. Taken together, while aging-related stressors help to elicit disease phenotypes in iPSC-derived models, a more complete induction of cellular age in iPSC-derived models seems favorable and represents a current focus in the field^58,59^.

#### iPSC models of premature aging syndromes

Human progeroid syndromes can provide additional insights into the mechanisms that control biological aging also during chronological aging (Fig. 1). iPSCs have been generated from patients suffering from Werner syndrome, Cockayne syndrome, xeroderma pigmentosum, ataxia telangiectasia, Fanconi anemia, and dyskeratosis congenita, all of which have defective DNA repair systems, and also from patients having alterations in the nuclear envelope such as HGPS (reviewed in^47^). In the context of this perspective, we will highlight the case of HGPS^28,29^. The extensive cardiovascular pathophysiology of HGPS ultimately contributes to most patients dying of myocardial infarction, heart failure, or stroke^60^, despite lacking major risk factors for cardiovascular disease (CVD)^6^. Interestingly, progerin accumulation is also observed in some tissues/cells during physiological aging (in living models without progeria) which makes this syndrome relevant for aging modeling^61^.

iPSCs derived from HGPS do not show a disease phenotype in their pluripotent state^9,62^ suggesting that the disease phenotype is only induced upon differentiation and LMNA expression. Thus, iPSCs derived from HGPS provide a unique approach to study how the cells get old besides being a source of aged cells. HGPS iPSCs have been used to derive smooth muscle cells (SMCs)^9,62–64^, endothelial cells (ECs)^64–66^, skin cells^67^, neural cells^40,68^, MSCs and their progenies^69–74^. In all these cellular models, several age-associated markers have been reported, including a decrease in cell proliferation^64^, increase in DNA damage^40,64^, abnormalities in nuclear morphology^9,40^, high expression of senescence-associated genes/proteins^9^, increased SA-β-galactosidase^63,64^, up-regulation of inflammatory cytokines^63,64^, shortened telomeres^64^, among others.

HGPS iPSCs were paramount to investigate molecular mechanisms of aging^40,67,75^. The first model of induction of cellular age in iPSC-derived neurons was largely inspired by HGPS, where overexpression of progerin in iPSC-derived neurons from PD patients resulted in cells that displayed age-dependent disease phenotypes^40^. When compared with wild type, HGPS-derived neurons exhibited a reduction in dendrite length, an increase in the expression of cleaved caspase-3 and a reduction in phosphorylated AKT, being the latter two associated with decreased neuronal survival^40^. HGPS-iPSCs may be also relevant for modeling skin aging, because HGPS individuals suffer from dermal hypopigmentation, a phenotype that is also common in the elderly^67^. Although melanocytes derived from HGPS-iPSCs did not exhibit changes in the expression of critical melanogenic markers, compared to their wild-type counterparts, they presented a reduction in melanin content and a decrease in melanosome maturity due to the accumulation of progerin^67^.

HGPS iPSC models have also been used as screening platforms to evaluate the mechanism and effects of pharmacological compounds that may be relevant both in the context of pathological as well as chronological aging. These models have been initially tested against drugs used in clinical trials^71,76^ and then against novel therapeutic drugs^9^. MSC derived from HGPS-iPSCs have been used to screen drugs, such as rapamycin^76^ and metformin^71^, able to reduce progerin accumulation^70,71,76^. Interestingly, the activation of retinoic acid (RA) signaling in MSCs by RA receptor agonists can decrease the expression of lamin A and progerin expression, while antagonists can increase their expression^72^. Importantly, the effect of RA agonists or antagonists is quite dependent on the microenvironment stiffness: stiffer substrates lead to a higher accumulation of RA receptor G isoform in the nucleus, culminating in an increased sensitivity of MSCs to RA signaling^72^. This observation suggests that both soluble and insoluble factors present in the microenvironments (which are highly dependent on the age of an individual and the type of tissue) can be determinants for the phenotype observed in progerin-expressing adult stem cells and on the efficacy of putative pharmacological treatments.

Overall, despite the usefulness of premature aging syndrome iPSC models, the separation between pathological and physiological aging in cellular models remains a challenge, and further investigation is required to clarify this issue (Fig. 1).

### Direct transdifferentiation to preserve endogenous cellular aging features

**Fig. 2:**
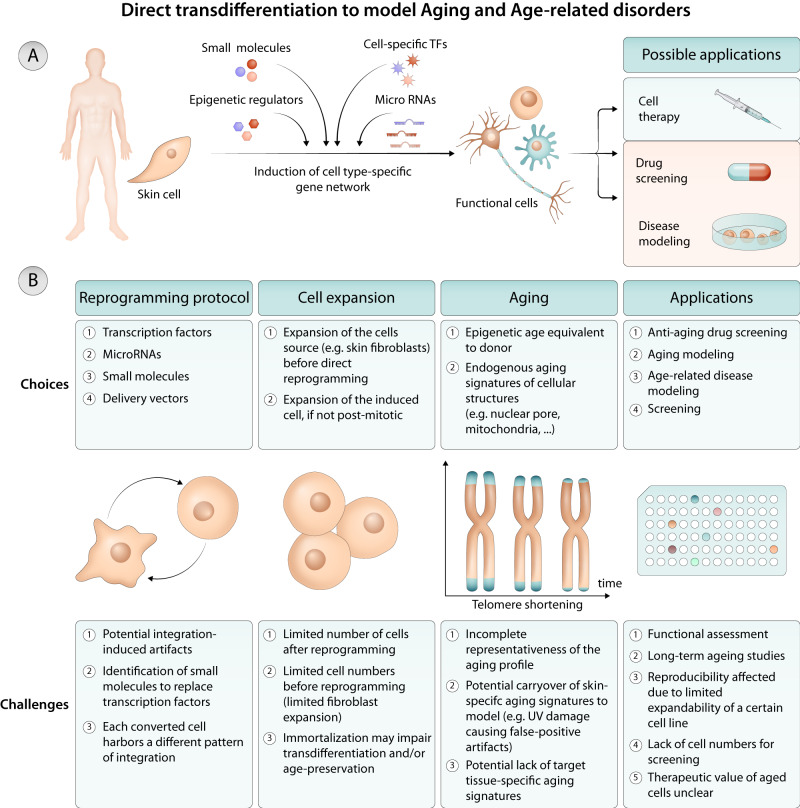
Modeling human aging with direct transdifferentiation of somatic cells. **A** Direct transdifferentiation of skin cells (easy available) into somatic cells that are not easy to isolate (e.g. neurons, cardiomyocytes, etc.) can be achieved by reprogramming with cell-specific transcription factors, microRNAs, small molecules, epigenetic regulators, etc. The derived cells can be used for cell therapy, drug screening and disease modeling. **B** Choices and challenges in the use of transdifferentiated cells for human aging modeling.

A limitation in using iPSC-derived cells for aging research, and in particular for modeling age-dependent disorders is the fact that they are rejuvenated early embryonic-like cells that do not endogenously exhibit cellular aging features. For such studies, the direct cellular transdifferentiation from one somatic cell type to another, without undergoing an intermediary pluripotent or progenitor cell type, represents an alternative strategy. Directly transdifferentiated, or converted, cells broadly retain aging-associated features of the original somatic cells, and may be used to study aging^77–79^ (Fig. 2). Conceptually, transdifferentiation is comparable to iPSC reprogramming, in that lineage-determining pioneer transcription factors, often in combination with microRNAs, and signaling pathway modulators, facilitate cell type change^80–82^. In most cases, dermal fibroblasts are used as a starting cell type, and the timeline for direct transdifferentiation ranges between a few weeks to two months, and is thus comparable to differentiation protocols of iPSCs (once iPSC lines are established). Transdifferentiated cells are polyclonal cultures, originating from different fibroblasts in the culture, while iPSCs are clonal cells, each derived from a single founding fibroblast or blood cell. Therefore, transdifferentiated cells not only reflect interindividual variability but also capture the genetic mosaicism of the fibroblast culture of each patient, which may be advantageous in modeling conditions such as aging. Using such approaches, wild type^83,84^ or disease (PD, AD, Huntington´s disease, HGPS) fibroblasts^12,13,79,85,86^ have been transdifferentiated into induced neurons (iNs)^12,13,15,85,86^, astrocytes^16,84^, endothelial^79^, smooth muscle cells^79^ among others. For example, striatal medium spiny iNs directly reprogrammed from fibroblasts of patients with Huntington’s disease (HD) showed alterations in chromatin accessibility^85^, degeneration by impaired autophagic function^85^ and mitochondrial dysfunction^86^ in an age-dependent and repeat extension-dependent manner. HD patient iNs showed mutant huntingtin aggregates, DNA damage, spontaneous neuronal death, decline in mitochondrial function, aging-related changes in chromatin accessibility, and impaired autophagy^85–88^. Further, and in contrast to dopaminergic neurons derived from iPSCs generated from PD fibroblasts, dopaminergic iNs generated from the transdifferentiation of adult human dermal fibroblasts isolated from idiopathic PD patients endogenously showed altered autophagy, increased DNA damage and increased accumulation of phosphorylated α-synuclein^13^. PD-patient iNs exhibited lower basal chaperone-mediated autophagy compared with healthy donors and in stress-induced autophagy and they displayed an age-dependent accumulation of macroautophagic structures^13^. Sporadic AD patient-specific iNs showed markers of oxidative stress, DNA damage and destabilization of epigenetic neuronal identity programs^12^. These aging AD patient-based cellular models further showed that in AD, neurons have lost specialized neuronal functions and resilience to control cells^12^ and develop senescence and pro-inflammatory phenotypes that can compromise surrounding cells via bystander effects^15^.

Although cellular transdifferentiation offers an elegant strategy to generate aged cells, this strategy also has limitations. One of the primary limitations of direct conversion models is the finite number of starting material, as the method does not involve expandable stem cell stages. This results in lower cell numbers, which poses challenges for scaling up the system for material-demanding molecular assays or screening approaches that are very doable with iPSC-based models^77,82^ (Fig. 2). The reprogramming potential of fibroblast cell lines into other types of cells is largely contingent on the quality of the fibroblast culture. While the generation of high-quality fibroblast cell lines from skin punch biopsies is a routine practice in many labs and clinics today, some patient cell lines are exceedingly rare. In cases where existing fibroblast lines are of low quality or become depleted, the procurement of new biopsies may be necessary, which is not always feasible. In addition, the preservation of only skin-related signatures that are encoded in the starting fibroblasts (e.g., if they relate to sun exposure of the skin) are likely present also in transdifferentiated cells. This may cause artificial ‘false-positive’ aging signs in the transdifferentiated cells. In contrast, once an iPSC line is established, it is essentially immortal, providing a virtually perpetual cell source. The lower potential for genetic manipulation is a notable disadvantage of transdifferentiated cells compared to iPSCs, as iPSCs are very amenable to genetic engineering using CRISPR-based technologies to generate for example isogenic control cell models^9^. However, all CRISPR-related technologies including Cas9, Prime editing, and Base editing critically depend on the cell-intrinsic DNA damage repair mechanisms, and these tools are much less well-established in non-stem/-tumor cells such as fibroblasts and neurons, and their potentials are more limited due to the slow-growing and post-mitotic nature of these cells^89,90^.

## 2D versus 3D aging models

### 2D aging models

The majority of in vitro models for aging and age-related disorders make use of 2-dimensional (2D) cell cultures, which is mainly because they are: (i) relatively inexpensive, (ii) well established in cell biology research and (iii) easy to analyze using several molecular, biochemical and image-based assays. For example, such 2D culture systems were useful to investigate the mechanisms underlying the degeneration of aged HGPS-iPSC SMCs^91^, the aging program of iPSC-CMs cultured for extended periods in vitro^42^, the effect of ECM in the performance and induction of an aging program in human iPSC-derived CMs^11^, the maturation and drug response of aged hiPSC-derived neuronal networks^43^, the effect of substrate topography in inducing cell aging^92^ and the impaired function and high mechano-sensitivity of HGPS-iPSC ECs mediated by an increase in the activity of a transient receptor potential channel^65,93^. The scalability of 2D systems make them a useful system for large-scale drug testing. In addition, some morphological studies and imaging studies are easier in 2D than in 3D. Yet, 2D cell cultures have several limitations because the interaction with the underlying substrate prevails over the cell-cell and/or cell-ECM interactions, the apical-basal polarity is changed, cell migration is not constrained and the stiffness of the plastic dishes is not physiological ultimately impacting cell proliferation, migration and activity^94,95^.

### 3D aging models

#### Tissue-engineered models

To model age-related changes in cell-cell interactions, 3D cell cultures are believed to better recapitulate the physiology of an aged organ/tissue. 3D tissue models containing different cell types allow for the investigation of how cellular interactions might be affected by the presence of aged cells. Further, hetero-chronic cell models, i.e. mixing both aged cells with non-aged cells, has the potential to better understand how cell-cell communication might be involved in the aging process of a tissue and to evaluate the importance of the ECM components in regulating (enhancing or reverting) cellular aging. For example, it is known that senescent cells communicate with proliferative cells via the senescence-associated secretory phenotype (SASP) that includes a temporally-regulated moiety of soluble factors such as interleukin-1a, IL-6, IL-8, transforming growth factor β, extracellular vesicles, lipid mediators^96^. These factors can induce a senescence phenotype in proliferative cells^97^ as well as induce ECM remodeling^96^. By recapitulating some of the complex microenvironment found in living tissues, 3D culture systems comprising defined cell types and ECM components might be a better model for studying intercellular communication, ECM remodeling by aged cells and the impact of biomechanical forces in cellular activity.

Recently, studies have investigated the impact of 3D culture systems to study cell aging biology. Pseudo-3D systems, i.e. systems that are in the transition between a 2D and a 3D culture system but do not have the complexity of a 3D culture system, have been used to investigate in vitro single or multiple physiological parameters in aged cells. For example, aged cells (HGPS-iPSC SMCs) were cultured in microfluidic systems, under flow conditions, to investigate their response to biomechanical forces^9,63^. Under biomechanical stress, HGPS-iPSC SMCs displayed an exacerbated inflammatory profile which was reversed by pharmacological treatment with inhibitors of the enzyme hydroxymethylglutaryl-coenzyme A (lovastatin) or farnesyl transferase (lonafarnib)^63^. Another example of a pseudo-3D system was based in the co-culture of ECs with HGPS-iPSC SMCs in a microfluidic system, under flow conditions. It was demonstrated that HGPS-iPSC SMCs were vulnerable to flow shear stress and detached from the substrate by the action of matrix metalloproteinase 13 (MMP13)^9^. In other cases, a 3D culture system has been adopted to create tissue engineered blood vessels (TEBVs)^8,98^. TEBVs comprised a perfused 3D tubular structure with an outer layer composed of HGPS-iPSC SMCs and an inner layer composed of HGPS-iPSC ECs or wild-type iPSC ECs. These 3D systems have shown reduced vasoactivity and medial wall thickening (through extracellular deposition), with HGPS-iPSC SMCs having increased calcification and increased detachment relative to control cells. In a separate study, the direct neuronal reprogramming of AD patient fibroblasts, cultured in a 3D hydrogel, by miRNAs was able to capture age-related neuropathology and the interplay between Aβ accumulation, tau dysregulation and neuronal death^99^.

Overall, these tissue-engineered models are very promising to recapitulate in vitro aging profiles that are dependent on intercellular communication, ECM and biomechanical forces. Significant advances in this area are expected in the coming years due to the need for better understanding aging in a more complex network and because of the progresses in tissue/organ printing technology that allows the preparation of 3D tissues with high resolution.

#### Organoids

**Fig. 3:**
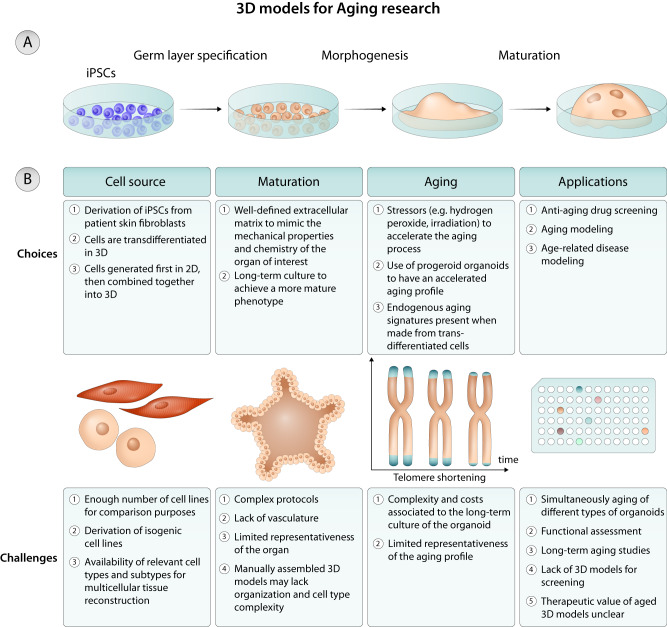
Modeling human aging with 3D models. **A** Creation of 3D aged models by organoids. Initially, iPSCs are differentiated into organoid-initiating cells followed by the culture of these cells in a 3D permissive ECM. **B** Choices and challenges in the use of 3D models for human aging modeling.

Organoids are three-dimensional multicellular cultures that mimic various features of an organ such as the structure and cellular composition^94,100,101^. Compared to conventional 2D cultures, organoids are complex 3D cellular systems can be originated from progenitor cells differentiated from pluripotent stem cells (or other sources^101^) and cultured in suspension or embedded in ECM. They rely in morphogenesis processes initiated by the organoid initiating cell (e.g. primary cell, tissue stem cell, or pluripotent stem cell) and, in some cases, a permissive ECM that allows cellular remodeling, a feature that sometimes is absent in tissue engineered organs (Fig. 3). These 3D cellular systems have the potential to provide near-physiological models to study human aging^101^. Organoids allow more degrees of freedom in long-term cultures, giving rise to cellular diversity, complex cell–cell interactions and unique physical structures. More recent advances in this area described the fusion of different organoids to form an assembloid^94^. For example, different organoids reflecting different brain regions or tissue are cultured together and allowed to form connections.

Recent studies have used organoids to study cellular aging^14,102,103^ and to identify treatments to ameliorate age-related diseases^104,105^. For example, brain organoids generated from iPSCs isolated from patients suffering from ataxia-telangiectasia (carrying a mutation in the ataxia-telangiectasia-mutated (ATM) gene that encodes the ATM kinase) have been used to investigate the impact of ATM kinase absence in the nervous system^14^. This is particularly relevant because the process of neurodegeneration in the disease is poorly understood and the animal models available fail to display clear symptoms of neurodegeneration. Brain organoids showed an increase of senescent cells located mostly within astrocyte populations, increased expression of pro-inflammatory genes as well as premature neuronal degeneration and dysfunction. Importantly, inhibition of cGAS and STING-dependent inflammation effectively suppressed self-DNA-induced SASP activation and rescued the neuropathological features in brain organoids. In a separate study, brain organoids generated from AD iPSC-derived cells or γ-irradiated brain organoids generated from wild-type cells, have been also used to screen drugs^104,105^. Moreover, aged colon organoids were obtained from human iPSCs to investigate whether DNA damage response was inversely correlated with an increase in the production of growth hormone^102^. Cellular aging was confirmed by an increase of p16, DNA damage, decrease of telomere length, increase in senescence-associated β-galactosidase activity and expression. Interestingly, aged colon organoids showed increased expression of growth hormone production at gene and protein levels and this production correlated with attenuated DNA damage response.

Although organoids offer the possibility to study human aging at the organ level, they have some limitations such as: (i) fail to completely recapitulate the aging profile at transcriptional and protein level, (ii) limited vascularization, (iii) low reproducibility and (iv) difficulty to stimulate the cells biomechanically^100,101^ (Fig. 3).

### Exploiting the power of human aging modeling: future directions

Although the field of human aging modeling in a dish by reprogrammed cells started more than 10 years ago with the use of iPSCs, it evolved for the use of direct conversion technologies and, more recently, for the use of tissue-engineered models and organoids. The number of studies describing the use of cellular transdifferentiation remains limited, mainly in that they focused mostly in the direct conversion of patient fibroblasts to neuronal cells^12,13,15,85,86^. It is expected that in the near future similar strategies will be used for the derivation of post-mitotic cells such as CMs or glial cells, which will help to advance our understanding of the human aging process. Other progresses are also expected in the use of iPSC-derived cells or transdifferentiated cells for the generation of 3D models of aging. Given the development of direct transdifferentiation protocols to generate different cell types of the same tissue, such as neurons and astrocytes of the brain, also multicellular 3D models based on age-preserved tissue models become possible. It will be interesting to build multicellular age-equivalent models for aging and age-related disorders of the brain, but also use them to build heterochronic modeling paradigms, which can contain combinations of young and old cells from patient and control donors.

The use of these cells for the generation of tissue-engineered models or organoids is yet in early stages with the first studies reported in the last 5 years^14,63,98,102,103,105^. These 3D models may be important to unravel the role of insoluble cues, such as ECM composition and/or substrate topography in aging processes. Recent studies have shown that the stiffness^106^, as well as the composition of the ECM^11,107^, are important regulators of the aging processes. In addition, it will be important to evaluate the crosstalk between aged cells with proliferative cells in a 3D environment to dissect how aging can be propagated. Next, in vivo xenotransplantation approaches have been shown to overcome limitations related to necrotic cores, and incomplete vascularization and maturation of iPSC-derived organoids, which will become relevant for long-term organoid models of aging^108–110^. Further, 3D live cell bioprinting approaches may be relevant to produce multiple types of artificial organs and tissues. In this case, a 3D printer add, layer by layer, cells and biologics to achieve tissues with a well-defined and organized 3D structure^111^. More advanced 3D tissues/organs will allow for the development of screening platforms for the identification of anti-aging drugs and can provide models of human age-related diseases that cannot be recapitulated in animals.
